# A Novel Curcumin-Galactomannoside Complex Delivery System Improves Hepatic Function Markers in Chronic Alcoholics: A Double-Blinded, randomized, Placebo-Controlled Study

**DOI:** 10.1155/2018/9159281

**Published:** 2018-09-23

**Authors:** Naveen T. Krishnareddy, Jestin V. Thomas, Saritha S. Nair, Johannah N. Mulakal, Balu P. Maliakel, I. M. Krishnakumar

**Affiliations:** ^1^Life Care Hospital, Bangalore, India; ^2^Leads Clinical Research & Bio Services Pvt. Ltd., Bangalore, India; ^3^R&D Centre, Akay Flavours & Aromatics Ltd., Kerala, India

## Abstract

Considering the recent interest in free (unconjugated) curcuminoids delivery, the present study investigated the efficacy of a novel food-grade free-curcuminoids delivery system (curcumin-galactomannoside complex; CGM) in improving the hepatic function markers (inflammation and oxidative stress) in chronic alcoholics. The double-blinded, placebo-controlled study randomized 48 subjects with elevated serum transaminases and gamma-glutamyl transferase (GGT) levels, who were allocated to two groups (n=24) and to receive either placebo or CGM at (250 mg × 2)/day for 8 weeks. While liver function markers (transaminases and GGT) in the placebo group showed an increase (~ 9.5%), CGM group indicated a significant decrease in transaminases (31%) and GGT (29%) from the baseline levels. The beneficial effect of CGM was also clear from the significant increase (p <0.001) in endogenous antioxidants (GSH, SOD, and GPx) and decrease in inflammatory markers (IL-6 and CRP) levels (p <0.001) as compared to both the baseline and placebo group. To summarize, the nutritional intervention of CGM-curcumin was found to offer a significant hepatoprotective effect to attenuate the alcohol induced alterations to hepatic function markers. The Indian Medical Council and Drug Controller General of India approved Clinical Trial Registry No. CTRI/2018/03/012385.

## 1. Introduction

World Health Organization (WHO) has reported Alcoholic Liver Diseases (ALD) as one of the world's leading causes of morbidity and mortality [1]. Alcoholism has been shown to have serious impact on the quality of life of even young population with loss of their productive life years [2, 3]. It is causative to more than 200 diseases and identified as the basic reason for almost 6% of deaths worldwide [1, 3]. Liver, being central to all the metabolic functions of body, has been found to be the organ mostly affected by alcohol and its toxic metabolites [4]. Chronic use of alcohol can damage various types of liver cells and can lead to the pathogenesis of liver diseases ranging from fatty liver to alcoholic hepatitis and further to cirrhosis and cancer [3, 5].

Ethanol mediated activation of CYP2E1 is an important event in the generation of free radicals and henceforth causing oxidative stress, inflammation, and cell damage [4, 6]. Alcohol has also been shown to disrupt the intestinal barrier with permeability to macromolecules like bacterial endotoxins (lipopolysaccharide) to elicit inflammatory cascades [4]. Metabolism of alcohol to more toxic metabolites such as acetaldehyde has been shown to promote the pathogenesis of fatty liver or steatosis by stimulating the synthesis of fatty acids and collagen in myofibroblasts [4, 5]. Fatty infiltration in hepatocytes has been shown to be enhanced by alcohol [6]. Thus, the development of steatosis and its further progress into chronic liver diseases involve complex interactions between the direct and indirect effects of alcohol and its metabolites [7].

Elevation in serum transaminases [alanine aminotransferase (ALT), aspartate aminotransferase (ALP)], and GGT (*γ*-glutamyl transferase) has been associated with chronic alcoholism [8]. While elevated serum transaminases was generally considered as an indication of liver diseases, elevation in alkaline phosphatase (ALP) in conjunction with aminotransferases indicates obstructive damage (cholestasis or blockage of bile flow) [9]. Elevation in GGT, a hepatic microsomal enzyme, is yet another marker for chronic alcohol induced structural liver injury and hepatic microsomal induction [10]. Though a number of synthetic drugs have been developed for the treatment of liver diseases, their long term use for the purpose of liver function management has been shown to cause side effects. Thus, the practice of safe botanical agents with proven antioxidant and anti-inflammatory effects has been widely accepted as a solution for the management of alcohol induced diseases [11].

Curcumin [1,7-bis(4-hydroxy-3-methoxyphenyl)-1,6-heptadiene-3,5-dione], the yellow pigment and the bioactive principle of the well-known curry spice turmeric (*Curcuma Longa L.*), has already been demonstrated as a prophylactic agent possessing a multitude of health beneficial pharmacological activities with unique pleiotropic mechanism of action and safety [12, 13]. But the poor oral bioavailability of curcumin contributed by its insolubility, instability, poor absorption, and rapid biotransformation has made it a typical class IV BCS molecule with poor pharmacokinetics [14, 15]. A number of enhanced bioavailable formulations of curcumin as demonstrated by the enhanced plasma concentration of curcumin metabolites (glucuronide and sulfate conjugates of curcuminoids and their reduced forms) have been reported and are even available commercially [16–19]. However, recent studies demonstrated poor membrane permeability and lack of anti-inflammatory, antiproliferative, and antioxidant activities of curcumin glucuronides, the major metabolites of curcumin [15, 20–23]. Thus, the oral delivery of free (unconjugated) curcumin over curcumin glucuronides is of great interest recently. The present randomized controlled study investigated the effect of curcumin on alcohol-induced hepatotoxicity for the first time. A food-grade formulation of natural curcuminoids [curcumin, demethoxycurcumin (DMC), and bisdemethoxycurcumin (BDMC)] as ‘curcumin-galactomannoside complex' (*hereinafter referred to as “CGM”*) that has been shown to possess enhanced oral bioavailability and cellular distribution of free forms of curcuminoids was employed in the present study. The study was based on the hypothesis that the bioavailability of free curcuminoids in CGM formulation could potentially amplify the beneficial effects of curcumin in chronic alcoholism induced elevation in hepatic function markers, oxidative stress, and inflammation. 

## 2. Materials and Methods

### 2.1. Material

Two-piece hard shell gelatin capsules of CGM (250 mg × 1) containing 39.1% curcuminoids, manufactured in a GMP (good manufacturing practices) facility, were obtained from M/s Akay Flavours & Aromatics Pvt. Ltd., Cochin, India. Total curcuminoids content as a sum of curcumin, DMC, and BDMC was estimated by a validated high performance liquid chromatography (HPLC) method as reported earlier [23]. Shimadzu M20 model HPLC fitted with photodiode array (PDA) detector at 420 nm (Shimadzu Analytical India Pvt. Ltd., Mumbai, India) and reverse-phase C18 column (250 × 4.6 mm, 5 *μ*m) (Waters India Pvt Ltd., Bangalore, India) was employed for analysis. Analytical reference standards of curcumin (CAS# 458-37-7; purity >98%), DMC (CAS# 22608-11-3; purity >98%), and BDMC (CAS# 33171-05-0; purity >95%) were obtained from Sigma-Aldrich, Bangalore, India. High pressure thin layer chromatography (HPTLC) was carried out in the HPTLC system (Camag, Muttenz, Switzerland) consisting of a development chamber with twin trough chamber (10 × 20 cm) and visualized using CAMAG TLC Scanner (Visualizer_171217). Densitometric analysis of the data obtained was carried out using winCATS software. Identical hard shell gelatin capsules of microcrystalline cellulose were used as placebo.

### 2.2. Study Subjects and Design

Healthy male subjects, aged between 30 and 50 years (n = 48), habitually consuming more than six units per week [1 unit =150 mL of wine or 360 mL of beer or 45 mL of 40% (v/v) alcohol] were enrolled from the outpatients list of Life Care Hospital, Bangalore, India, where the subjects visited for routine medical checkup and consultation. The selection was based on the criteria of National Institute on Alcohol Abuse and Alcoholism (NIAAA) for chronic alcoholism. As per NIAAA, a drinking pattern that brings blood-alcohol concentration (BAC) levels ≥ 0.08 g/dL is considered as chronic alcoholism [24]. We estimated the BAC levels of subjects using validated Widmark formula [25, 26] and found that a person consuming more than six units of alcohol per week would have a BAC of > 0.13 g/dL, meeting the chronic alcoholism criteria. The subjects underwent a standard clinical assessment comprising structured diagnostic interview on demographic characteristics, health conditions, hematology/ biochemical parameters, blood sugar, and blood pressure (Table 1). The study was carried out in accordance with the Indian Council of Medical Research (ICMR) regulations with Clinical Trial Registry, Government of India (CTRI) registration. The protocol was approved by a registered independent ethical committee in India (Reg. No.: ECR/184/Indt/KA/2014) and the study was performed in the premises where the affiliation of the committee and its jurisdiction is applicable.

Details of the inclusion and exclusion criteria are given in Table 2. Only healthy subjects requiring no immediate medical intervention, yet volunteering to take nutritional supplements to improve liver conditions, were chosen for the study. The most important inclusion criteria included chronic alcoholics with elevated liver function markers. Subjects abstaining from alcohol for more than 1 month were avoided from the study. Those diagnosed with any kind of liver diseases or associated conditions were also excluded from the study. Subjects were also advised to avoid any kind of herbal or other supplementations during the course of the study. Written consent from all individuals was obtained prior to the study and was assigned with a three-digit, unique randomization code.

### 2.3. Sample Size and Randomization

**Figure 1:**
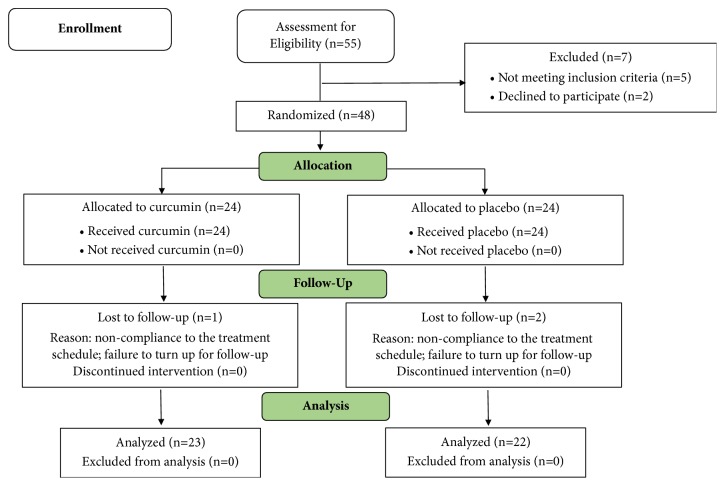
CONSORT flow diagram of the study.

The study was conducted in a randomized double-blinded, placebo-controlled manner as depicted in Figure 1. The sample size was presumed to be 53 for a population of 100 with the statistical significance level of 0.05. The selected subjects were randomized by allocating randomly permuted blocks, employing the computer generated allocation table (http://www.randomization.com).

### 2.4. Participants

Among the 55 male subjects initially found to meet the inclusion and exclusion criteria, two subjects refused to participate and five were found unfit for the study. Final sample selected was that of 48 subjects. Of these, two from placebo group and one from CGM group were lost during intervention. Hence, the final sample size was 22 in placebo and 23 in CGM group.

### 2.5. Study Design

Among the eligible and willing candidates, forty eight subjects were randomized to two groups to receive either CGM (n = 24) or placebo (n = 24) for 8 weeks. The study constituted four visits, including screening (Visit 1), baseline visit at day 0 (Visit 2), visit on 28th day (Visit 3), and visit on 56th day (Visit 4). During visit 1, selected candidates were randomized on the basis of their liver function tests and their written consent was obtained. During the baseline visit at Day 0, the selected participants were advised to report to the clinical laboratory of the hospital under fasting conditions for hematological/biochemical analysis. The subjects were then provided with standard breakfast consisting of a cup of tea/coffee or water and a typical south Indian vegetarian breakfast made of rice and vegetable curries having an approximate nutritional composition of fat, 22 to 24%; carbohydrates, 35 to 40%; protein, 27 to 30%; and energy, 400 to 500 calories. The same protocol was repeated on 56th day (visit 4) for the blood analysis. In this visit, the participants were also asked to update the medical history and anthropometric measurements. Sealed and coded high density polyethylene bottles containing sixty capsules of either CGM or placebo (250 mg × 1) with identical color, size, and shape were provided to the subjects and asked to record the pill consumption every day. Participants were also instructed not to change any lifestyle habits throughout the study, by maintaining habitual diet and physical activity. However, subjects were not allowed to consume more than three units of alcohol per week. The subjects were monitored on a weekly basis through telephonic follow-ups.

### 2.6. Blood Sampling and Biochemical Measurements

Fasting blood samples were collected from median cubital vein. Hematological parameters including red blood cell count (RBC), total and differential white blood cell count (WBC), platelet levels, and hemoglobin (Hb) content were determined using a hematology analyzer (Model-Diatron, Wein, Austria). Blood samples were centrifuged for 10 min at 11950* g *to separate the serum and stored at −80°C for analysis. Total cholesterol (Tc), low-density lipoprotein (LDL), high-density lipoprotein (HDL), triglycerides (TG), ALT, AST, and ALP levels were analyzed by assay kits provided by M/s Agappe Diagnostics Pvt. Ltd., Bangalore, India. Antioxidant status was measured by estimating serum levels of superoxide dismutase (SOD) activity [27], glutathione peroxidase (GPx) activity [28], and glutathione (GSH) activity [29]. Lipid peroxidation was determined by the analysis of thiobarbituric acid reactive substances (TBARS) by the method of Ohkawa et al. [30] and expressed as nmols/mL. GGT was assayed as described by Persijn and van der Slik [31]. Serum concentrations of C-reactive proteins (CRP) were measured with human specific ELISA kit (Invitrogen™ Novex™ from Life Technologies, Carlsbad, California, USA) following the manufacturer's instruction manual. All assays were performed in triplicate or more and the average value was reported. Serum IL-6 was measured using a commercial immunoassay (MEDGENIX IL-6 EASIA, Biosource Technologies, Inc., Europe S.A., Fleunes, Belgium).

### 2.7. Statistical Analysis

Statistical analyses were performed using one-way analysis of variance (ANOVA). Intergroup comparisons were performed using independent samples* t*-test (for normally distributed data). The differences were considered significant at p < 0.05. Reported values are arithmetic means with standard deviations (SD) or standard errors of the mean (SEM) as indicated. 

## 3. Results

### 3.1. Subjects and Study Material

The average age of the participants in the placebo group was 38.6 ± 3.92 years and that of the treatment group was 40 ± 3.1 years, respectively. Baseline anthropometric, body composition, and blood pressure characteristics of both CGM and placebo groups were comparable (Table 1). Among the 48 subjects grouped into equal sizes of placebo and CGM groups (n = 24), there was a dropout of two subjects from the placebo and one subject from the CGM group. None of the dropouts was due to the side effects but due to the noncompliance with the treatment regime, mainly the difficulty to control alcohol consumption. Biochemical analysis revealed identical levels of liver function markers (AST, ALT, and ALP) between the groups, with no statistically significant difference (p >0.05). Other hematological and biochemical clinical markers were within the healthy ranges in both the placebo and CGM groups at the baseline (Table 1).

**Figure 2:**
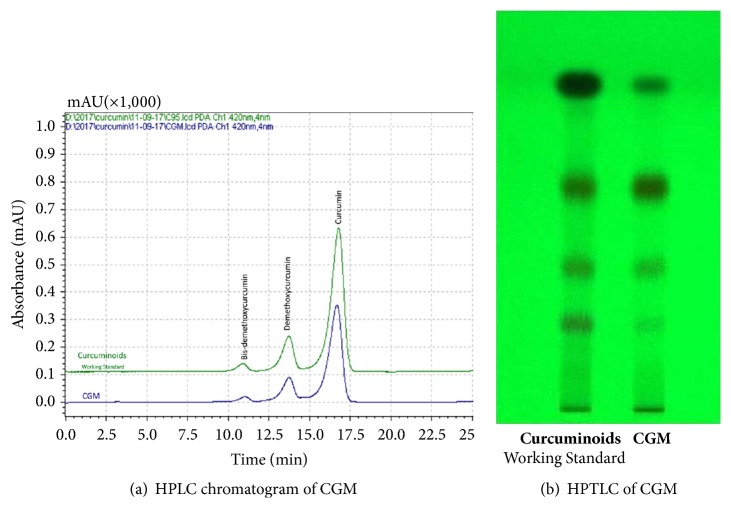
HPLC and HPTLC analysis of CGM in comparison with standard curcumin.

The protocol used in the present study is depicted in Figure 1. Identical hard shell gelatin capsules (250 mg ×1) of both CGM and placebo were obtained along with detailed certificate of analysis. Both HPLC and HPTLC analyses were performed for the identification, confirmation, and quantification of curcuminoids (Figure 2). The mobile phase for HPLC consisted of 43:57 (v/v) of acetonitrile, water containing 0.2% phosphoric acid, and that for HPTLC was chloroform, methanol (48: 2) (v/v). It was found that CGM contains a total of 39.1% curcuminoids with a relative distribution of curcumin, demethoxycurcumin (DMC), and bis-demethoxycurcumin (BDMC) in the ratio of 77.4, 14.7, and 3.2%, respectively. Microcrystalline cellulose (MCC) colored with approved food color, to look like curcumin, was employed as the placebo.

### 3.2. Effect of CGM on Liver Toxicity Markers

**Figure 3:**
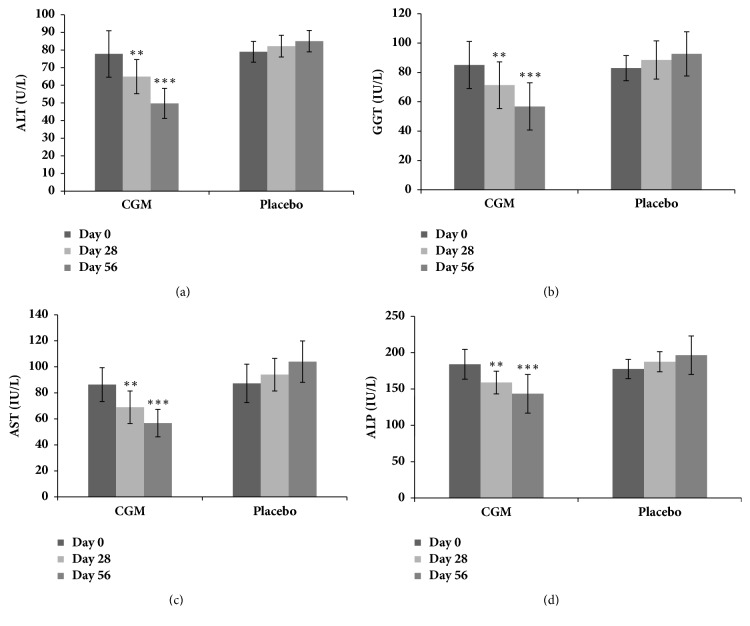
Liver toxicity markers in serum;* significant difference in p values given as ∗∗ = p < 0.01; ∗∗∗ = p < 0.001, and ns = p > 0.05, when the CGM group is compared with that of placebo.*

Liver function marker enzymes (ALT, AST, ALP, and GGT) were measured on Day 0 (the baseline), Day 28 (Visit 3), and Day 56 (Visit 4). At the baseline, all the subjects (both in placebo and in CGM groups) had significantly (p <0.01) higher levels of liver function markers as compared to the generally accepted safe limits. There was no significant variation (p >0.05) between the placebo and CGM groups (Figure 2). During 56 days of study period, the placebo group showed an increase in hepatic function markers from Day 0 to Day 28 and further to Day 56, as a characteristic feature of fatty liver conditions. However, a significant (p <0.001) decrease in these markers was observed among CGM group. The transaminase ALT, one of the most sensitive liver damage marker, while exhibiting an increase of 4.02% from the baseline by Day 28 and 7.8% by Day 56, the same was found to be reduced by 16.5% by Day 28 and 36% by Day 56 (p < 0.001) for CGM group (Figure 3(a)). Yet another alcoholic liver damage marker GGT was observed to elevate by 6.75% to 11.8% (p >0.05) from Day 28 to Day 56 in placebo. But the same was reduced by 16% on Day 28 and further to 29% by Day 56 (p <0.001) (Figure 3(a)). The other markers, AST and ALP, also showed a similar pattern of gradual increase from Day 0 to Day28 and Day 56 in the placebo group. But these too were also brought down significantly in CGM group (p <0.01) (Figures 3(c) and 3(d)). Thus, intragroup comparison showed an average of 32% decrease from the baseline liver function markers in CGM group, as compared to an average increase of 13.4% in placebo. Intergroup comparison on the other hand also indicated almost 38.6% decrease in CGM group as compared to the placebo.

### 3.3. Effect of CGM on Oxidative Stress Markers

**Figure 4:**
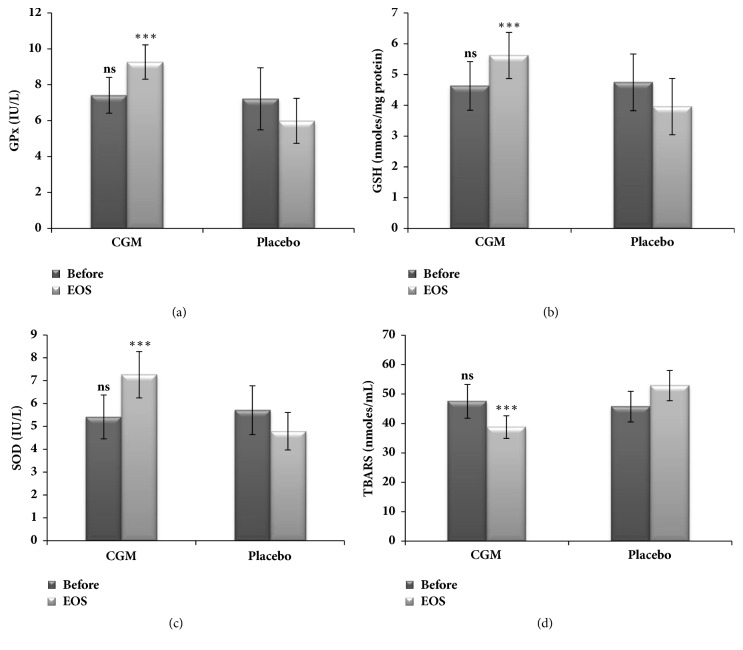
Oxidative stress markers in serum;* significant difference in p values given as ∗∗ = p < 0.01; ∗∗∗ = p < 0.001, and ns = p > 0.05, when the CGM group is compared with that of placebo.*

Endogenous antioxidants (GPx, GSH, and SOD) and lipid peroxidation were found to be significantly varied in both the placebo and CGM groups during the study period of 8 weeks (Figure 4). While the endogenous antioxidants GSH and SOD showed a significant enhancement (p <0.001) upon supplementation of CGM, the plasma levels of lipid peroxidation were found to be significantly (p <0.01) decreased from the baseline. Ethanol associated depletion of GSH was reverted by 19.6% in CGM group (p <0.001), as compared to the placebo which showed a decline of 21.2% from the baseline (Figure 4(a)). The average enhancement in SOD activity in CGM group was 23.4% from the baseline (p <0.001), as compared to the decline of 11.3% in placebo by the end of the study period (Figure 4(b)). GPx activity also declined by 17% by Day 56 in placebo subjects. But, by Day 56, CGM effectively reverted this condition by 24.8% (Figure 4(c)). The extent of lipid peroxidation is indicated by the elevation in TBARS values which was maximum in the placebo as compared to CGM group. TBARS levels were lowered by 18.9% in CGM group (p <0.001) whereas the placebo showed an increase of 12% (Figure 4(d)).

### 3.4. Effect of CGM on Inflammatory Markers

**Figure 5:**
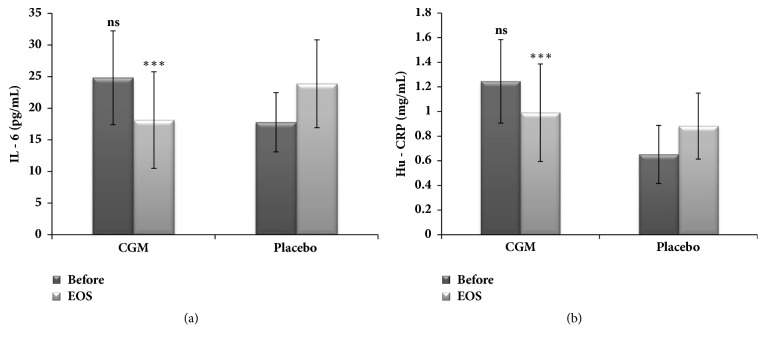
Inflammatory marker in serum;* significant difference in p values given as ∗∗ = p < 0.01; ∗∗∗ = p < 0.001, and ns = p > 0.05, when the CGM group is compared with that of placebo.*

Average baseline values of the serum levels of IL-6 and CRP in both placebo and CGM groups were different (p >0.05). During 56 days of the study period, both IL-6 and CRP decreased significantly (p <0.01) and progressively from Day 0 through Day 56 in CGM group, whereas a progressive increase was observed (p <0.01) in placebo (Figure 5). The increase in CRP level in the placebo group was 34% by the end of the study, indicating a steady increase in inflammation (Figure 5(a)). Upon CGM supplementation, CRP level was decreased by 27.3% from baseline (Figure 5(a)). In the case of IL-6, the increase in the placebo group by the end of the study period was 16.1% as compared to a 15.1% decrease in CGM group (Figure 5(b)), which was statistically significant (p <0.01) with respect to both inter- and intragroup comparisons.

## 4. Discussion

Chronic consumption of alcohol was shown to elicit cascades of inflammatory and oxidative stress responses, responsible for the structural and functional changes in various liver cell types leading to hepatotoxicity and liver damage [7]. Damaged liver can seriously affect the metabolism, detoxification process, and synthesis of micronutrients which play vital role in the maintenance of health [32]. Alcohol and acetaldehyde have been shown to activate Kupffer cells which in turn activate TNF-*α* and stimulate NF-*κ*B, a transcriptional factor that triggers the induction of inflammatory genes [4]. Enhanced lipid peroxidation and significant elevation in liver function marker enzymes were reported under conditions of fatty liver or steatosis [33]. Thus, the pathogenesis of alcohol-induced liver diseases represents a very complex mechanism involving overproduction of reactive oxygen species, oxidative stress, inflammatory events, and morphological changes corresponding to fat accumulation, hepatocellular injury, and necrosis [4].

Natural antioxidants and anti-inflammatory agents can play very significant role in the maintenance of liver health [11, 34]. The objective of the present study was to investigate the effect of curcumin on subjects with moderate alcoholic fatty liver characterized by significant elevation in liver function marker enzymes. Curcumin has already been demonstrated to have diverse molecular targets ranging between circulatory proteins, enzymes, and genes involved in inflammation, oxidative stress, and even apoptosis making it an ideal molecule for the development as a therapeutic agent and/or functional food ingredient [13, 35]. But the poor absorption, rapid biotransformation into various metabolites of relatively low activity, and lack of cellular uptake have been recently identified as the major problems for its therapeutic efficacy [36]. Thus, the present study employed a highly bioavailable form of curcumin, CGM, that has been shown to deliver bioactive forms of free (unconjugated) curcuminoids into plasma with significant cellular uptake when administered orally [37, 38]. It was hypothesized that formulations capable of delivering significant levels of free curcuminoids over conjugated curcumin metabolites would alleviate the earliest responses of chronic ethanol exposure such as elevation in liver function markers.

Considering the earlier human bioavailability study of CGM at 250 mg dose and its favorable pharmacokinetics with regard to the maximum absorption (C_max_), elimination half-life (T_1/2_), and area under curve (AUC) of free curcuminoids over relatively less reactive curcumin glucuronide metabolites [38], the present study employed a convenient dosage of (250 mg × 2)/day of CGM. Despite various* in vitro *and* in vivo* reports on hepatoprotective effects of curcumin, the present investigation is the first attempt to investigate its efficacy among alcohol induced fatty liver subjects. A recent clinical study has reported on NAFLD subjects indicating the positive effects of curcumin [39].

The biochemical markers that are widely studied among chronic alcoholics are serum GGT and transaminases [5]. Elevated serum transaminases were generally considered as an indication of liver disease and serum ALT and AST were most commonly assayed for screening and monitoring patients with liver conditions [35]. In the present study, supplementation of CGM was found to induce significant reduction in the elevated levels of serum transaminases. The observed effect was also found to be in agreement with the results of a previous study on subjects with NAFLD [39], suggesting the beneficial effect of curcumin on hepatic condition. Though the reduction in the present study was visible by Day 28 itself, it progressed to a more significant level by Day 56. Yet another important marker of alcohol-induced liver damage is GGT, a hepatic microsomal enzyme [5]. Elevation in GGT is a commonly used marker for chronic alcohol consumption and is an indicative of structural liver injury and hepatic microsomal induction [10]. While CGM group showed a significant reduction in GGT, placebo does not induce any reduction but rather offered an elevation in many subjects.

Alcoholism has been demonstrated to cause deviation in cellular homeostasis generating reactive oxygen and nitrogen species (ROS) and oxidative stress which further leads to the depletion of endogenous antioxidant defenses and induction of pathological conditions like hepatocellular dysfunction and loss of membrane integrity [33]. It has been reported earlier that GPx, SOD, and GSH were significantly reduced among alcoholics [40]. In agreement with these findings, the present study also observed significant decrease in the activities of SOD, GPx, and GSH with a concurrent elevation in the extent of lipid peroxidation in placebo group. But, CGM group exhibited an elevation in these markers from Day 0 to Day 56, indicating the antioxidant efficacy. A recent study on subjects characterized with occupational stress has also reported a significant enhancement in endogenous antioxidants of CGM group, as compared to unformulated standard curcumin supplemented subjects [41]. While GSH was established as an essential antioxidant involved in the detoxification process, other radical scavenging systems were also implicated for prevention of ROS-induced damage hepatocytes. When these antioxidant defense systems are rendered insufficient, as in the case of alcoholism, the administration of external antioxidants and scavengers was reported to have therapeutic potential [42].

Inflammation also plays a key role in the development of liver diseases and has been shown to be significant under conditions of steatosis [31]. Chronic alcohol consumption was found to induce significant inflammation with upregulation of proinflammatory cytokines and CRP leading to enhanced activity of matrix metalloproteinases (MMP-2 and MMP-9) and toll-like receptors (TLR4) [43]. CRP, an acute phase plasma protein synthesized within the hepatocytes, has been found to elevate under the transcriptional control of cytokine IL-6 during conditions of inflammation, cellular leakage, tissue damage, and necrosis in the liver parenchyma [44]. Elevated levels of IL-6 and CRP were reported among alcoholics infected with human immunodeficiency virus [45]. In the present study, a significant decrease in CRP and IL-6 was observed upon CGM supplementation. However, placebo group failed to produce any such apparent decline, indicating the ability of CGM to modulate the inflammatory responses.

## 5. Conclusion

The present randomized controlled study evaluated the efficacy of curcumin among subjects diagnosed to have alcohol induced fatty liver characterized by elevated levels of liver function marker. Considering the significance of free (unconjugated) curcuminoids in bioactivity, a novel bioavailable formulation of curcumin with fenugreek galactomannans (CGM) that has already been shown to possess enhanced bioavailability of curcuminoids was employed in the present study. We found CGM to be an effective nutritive intervention for chronic alcoholics through its efficacy to modulate oxidative stress, inflammation, and hence the elevation in liver function enzymes. However, relatively low sample size, lack of ultrasound sonography screening, tea/coffee intake of the subjects during the study period, and the absence of female subjects constituted the major short comings of the present work, which need to be addressed in future trials.

## Figures and Tables

**Table 1:** Comparison of baseline and anthropometric characteristics between CGM and placebo groups.

	**Initial**		**Final**	
	**CGM**	**Placebo**	**CGM**	**Placebo**
**Age**	45± 9.1			
**Gender**	Male			
**Height **(in cm)	165.7± 6.2			
**Body weight** (kg)	73.45±6.1	73.2±8.9	72.2±7.1	70.12±5.2
**BMI** (kg/m^2^)	31.2±3.6	30.10±5.0	30.8±3.5	29.88±4.5
**SBP** (mmHg)	124.41±3.2	126±3.4	121.3±3.4	124.7±3.3
**DBP** (mmHg)	83.3±6.4	86.23±5.1	82.52±4.8	84.54±2.7
**Hb** (g/dL)	13.2±1.16	13.4±0.88	13.5±1.19	13.0±0.70
**Creatinine **(g/dL)	1.18±0.2	1.1±0.2	1.1±0.2	1.09±1.8
**Cholesterol** (mg/dL)	245.2±30.2	243.8±30.3	234.3±20.1	258.2±32.7
**HDL** (mg/dL)	45.21±4.2	42.55±5.5	53.21±4.3	40.21±4.1
**LDL **(mg/dL)	109.2±21.5	112.7±17.5	100.4±15.1	120.7±18.1
**Triglyceride** (mg/dL)	199.5±49.5	189±28.6	176.2±42.7	191.3±34.2

SBP: systolic blood pressure.

DBP: diastolic blood pressure.

BMI: body mass index.

Hb: haemoglobin.

HDL: high density lipoprotein.

LDL: low density lipoprotein.

**Table 2:** Inclusion and exclusion criteria for the study.

**Inclusion criteria**	**Exclusion criteria**
Healthy subjects with chronic alcohol consumption	Subjects abstaining from alcohol for more than 1 month, concomitant symptomatic or asymptomatic bacterial infection and severe bacterial infection within the previous 3 months
Elevated liver function markers- Increased alanine aminotransferase (ALT), aspartate aminotransferase (AST), and alkaline phosphatase (ALP)] AST:ALT > 2	Subjects diagnosed with any diseases, and those with renal disorder, hepatitis (ALT level ≥ 3 times the upper limit), and gall bladder diseases or its removal, cardiovascular, pulmonary, kidney disease, pancreatitis, type I diabetes.
No evidence of liver diseases- no evidence of liver diseases due to viral hepatitis, autoimmune disease, hemochromatosis, Wilson's disease or drug-induced hepatitis	Subjects who are currently under any medications or herbal supplements.

## Data Availability

The data used to support the findings of this study are included within the article.
