# Corrigendum to “A Novel Curcumin-Galactomannoside Complex Delivery System Improves Hepatic Function Markers in Chronic Alcoholics: A Double-Blinded, randomized, Placebo-Controlled Study”

**DOI:** 10.1155/2019/5673740

**Published:** 2019-03-03

**Authors:** Naveen T. Krishnareddy, Justin V. Thomas, Saritha S. Nair, Johannah N. Mulakal, Balu P. Maliakel, I. M. Krishnakumar

**Affiliations:** ^1^Life Care Hospital, Bangalore, India; ^2^Leads Clinical Research & Bio Services Pvt. Ltd., Bangalore, India; ^3^R&D Centre, Akay Flavours & Aromatics Ltd., Kerala, India

In the article titled “A Novel Curcumin-Galactomannoside Complex Delivery System Improves Hepatic Function Markers in Chronic Alcoholics: A Double-Blinded, randomized, Placebo-Controlled Study” [1], the dosage of drugs in the Materials and Methods and Results was given incorrectly as (250 mg x 1)/ day. It should be corrected to (250 mg x 2)/ day.
